# Care Home Life and Identity: A Qualitative Case Study

**DOI:** 10.1093/geront/gny090

**Published:** 2018-08-04

**Authors:** Katie Paddock, Christine Brown Wilson, Catherine Walshe, Chris Todd

**Affiliations:** 1 School of Health Sciences, Faculty of Biology, Medicine and Health, Division of Nursing, Midwifery and Social Work, University of Manchester, UK; 2 Manchester Academic Health Science Centre, UK; 3 School of Nursing and Midwifery, Queen’s University, Belfast, UK; 4 International Observatory on End of Life Care, Division of Health Research, Furness Building, Lancaster University, UK; 5 Manchester University Foundation NHS Foundation Trust, UK; 6 Psychological Sciences, Institute of Psychology, Health, and Society, University of Liverpool, Liverpool, UK

**Keywords:** Institutional care/residential care, Qualitative analysis: case study, Qualitative research methods, Identity, Social identity perspective

## Abstract

**Background and Objectives:**

The transition to a care home can involve multiple changes and losses that can affect an older person’s well-being and identity. It is not clear how older people perceive and manage their identity within a care home over time. This study explores how living in a care home affects the identities of residents and how they address this in their daily lives.

**Research Design and Methods:**

A multiple qualitative case study approach incorporated interview and observational data. Eighteen semistructured interviews and 260 hr of observations were conducted over 1 year with care home residents, relatives, and staff across three care homes within Greater Manchester, UK. Data were analyzed using framework analysis, drawing on the social identity perspective as an interpretive lens.

**Results:**

Four themes were identified: (a) changing with age, (b) independence and autonomy, (c) bounded identity, and (d) social comparison. The impact of aging that initially altered residents’ identities was exacerbated by the care home environment. Institutional restrictions jeopardized independence and autonomy, provoking residents to redefine this within the allowances of the care home. Strict routines and resource constraints of well-meaning staff resulted in the bounded expression of personalities. Consequently, to forge a positive identity, residents without dementia engaged in social comparison with residents with dementia, emphasizing their superior cognitive and physical abilities.

**Discussion and Implications:**

Social comparison as an adaptive strategy has previously been unidentified in care home literature. Residents need more support to express their identities, which may reduce the necessity of social comparison, and improve interrelationships and well-being.

Moving to long-term residential and/or nursing care facilities (hereafter referred to as “care homes”) involves a series of changes that can affect an older person’s sense of identity (Froggatt, Davies, & Meyer, 2009; Næss, Fjær, & Vabø, 2016; Tajfel & Turner, 1979). Residents can become disconnected from facets or symbols of their identity, including social networks, familiar routines, recreational activities, and meaningful belongings. This disconnect can result in poor well-being or depression (NCHR&D, 2006; Tester, Hubbard, Downs, MacDonald, & Murphy, 2004). In addition, the transition to a care home often occurs at the nadir of physical and/or cognitive abilities (Kingston et al., 2017), thereby limiting residents’ functional abilities to adapt to this new context and increasing their reliance on care staff to facilitate identity maintenance. In England, supporting identities is a quality standard for care homes, but variations in care quality, limited resources, and poor workforce morale can impede such aims (Alzheimer’s Society, 2013; Care Quality Commission, 2016; Lievesley, Crosby, Bowman, & Midwinter, 2011). To improve residents’ sense of identity in care homes, we must understand how it is negotiated within this complex context.

Few studies have explored the daily impact of life within care homes on identity, particularly from the perspectives of relevant stakeholders; residents, their significant others, and care home staff. In this article, we address this gap. We use the social identity perspective (SIP) as a theoretical lens to explore the strategies that residents use to adapt to life in a care home over time, and the daily contributions of others in the co-construction of residents’ identities.

SIP holds that individuals’ overall sense of identity is a composite of memberships to meaningful social groups (social identity) and idiosyncratic personal attributes (personal identity). Identity maintenance is an inherently social process that occurs across the life course, where different identities come to the fore within different salient contexts (Hogg & Abrams, 1988; Oakes, Haslam, & Turner, 1994; Turner, 1982). Major life events, such as the transition to a care home, can disrupt connections to social groups and idiosyncratic attributes (Hockey & James, 2003; NCHR&D, 2006; Kroger, Martinussen, & Marcia, 2010; Tajfel & Turner, 1979). Maintaining social relationships or establishing new connections buffers against negative outcomes (A. Haslam, Jetten, Postmes, & Haslam, 2009; Jetten & Pachana, 2012), but studies have shown physical and interpersonal barriers to this (Abbott, Bangerter, Humes, Klumpp, & Van Haitsma, 2017; Hubbard, Tester, & Downs, 2003), limiting opportunities to bolster identities within this new context.

Social groups are also judged by others as being of a higher or lower status, and the positivity of one’s identity is derived from the internalization of these evaluations (Tajfel & Turner, 1979). Adaptive strategies can be used to maintain a positive identity when associated with a negatively perceived group (Reicher, Spears, & Haslam, 2010; Tajfel, 1981; Tajfel & Turner, 1979). These include (a) social mobility: physically or psychologically leave the group and adopt a different identity; (b) social creativity: reframing the negativity as something positive, changing comparator dimensions to something more positive, or changing the comparison group to an even more negatively perceived group; and (c) social competition: direct competition with the outgroup. The use of these strategies will depend on the perceived permeability of the boundaries between groups. SIP, therefore, emphasizes the social- and context-dependent nature of identity.

SIP has been used in other social care areas (Black et al., 2018; Iyer, Jetten, Tsivrikos, Postmes, & Haslam, 2009; Jetten & Pachana, 2012; Knight, Haslam, & Haslam, 2010), but it has been used much less frequently in care homes (C. Haslam et al., 2014) and with little focus on the social- and context-dependent nature of identity from multiple perspectives. In this study, we use SIP to explore identity management within the care home context and incorporate the perspectives of residents, their relatives, and staff members. This will help inform approaches for supporting residents to maintain a positive sense of self and improve well-being, and improve their experiences of long-term care.

## Methods

### Study Design

This study used a multiple qualitative case study approach. Case study methodology facilitates the triangulation of multiple methods and sources of evidence to explore complex, context-dependent phenomena (Walshe, Caress, Chew-Graham, & Todd, 2004; Yin, 2009), which is congruent with the inherently social and complex, context-dependent nature of identity management in SIP. Cases were defined as individual care homes. Within each case, data were collected using interview and observation methods (see below) to explore how daily life in a care home influences identity from multiple stakeholders’ perspectives.

The following theoretical propositions (Yin, 2009), based on SIP and care home literature, were used to guide data collection and analysis:

1. Residents will renegotiate their identities within the context of the care home in light of new social relationships and interactions;
2. Maintaining links with previous social networks and habits (e.g., daily routines, personal décor) will be important for residents to maintain a sense of self; and
3. The care home environment will have the potential to accommodate a multitude of identities with adequate support from individuals and appropriate resources.

### Within- and Cross-Case Sampling and Recruitment

Care homes in Greater Manchester, UK, were recruited through local research networks and via gatekeepers. Cases were purposefully sampled to vary in size (number of beds), location (high- or low-income areas), and building type (converted house or purpose-built facility). This aimed to acquire a broad range of experiences, and theoretical replication, where differing variables across cases are anticipated to yield contrasting results (Yin, 2009, 2010). The intended case sample was small to encourage rich, contextualized data, to understand the phenomenon under study (Cleary, Horsfall, & Hayter, 2014; Geertz, 1973). Twenty-three care homes were approached to participate, and three care homes agreed. Table 1 illustrates basic information about the care homes.

**Table 1.** Features of Participating Care Homes

Feature	Care Home 01	Care Home 02	Care Home 03
Number of residents (maximum)	17	37	28
Number of residents with capacity to consent (approximately over course of data collection period)	8	8	7
Type of care home	Residential care only	Residential care with nursing	Residential care only
Location	Low–medium income area	High-income area	Low-income area
Building type	Converted house	Converted house	Converted house

Care home residents, family and friends, and staff, who met the following inclusion criteria, were eligible to participate: Residents aged 65 years or older, who had capacity to consent; all staff who had regular contact with residents, including managerial and nursing staff; all visitors who were a relative or long-term acquaintance of a resident (collectively termed “relatives” for ease). Staff identified residents with capacity to consent. Only individuals who could speak English were included, although only one resident was excluded by this constraint.

Prior to study commencement, the first author (K. Paddock) spent an introductory period within each care home. She introduced herself and the study, and engaged in informal conversations, to ensure that potential participants were comfortable with her presence and identified her as a researcher, not a visitor or staff member.

Convenience and purposeful techniques were used to sample residents, their relatives, and staff for interview. These included if residents/relatives had particularly positive or negative experiences of the move to a care home and subsequent adjustment or staff who were involved in daily decision making in the care homes or care of residents. Informed consent was obtained prior to recording of interviews. It was not possible to obtain written consent prior to observations due to the busy, often transient nature of care homes, and the risk of disrupting daily care or altering the dynamic of any event being observed. Information about the study and observations were displayed in each care home, and before each observation, individuals were verbally made aware of the researcher’s presence. Individuals could opt-out of observations via the researcher, members of staff, or opt-out form, and any field notes would then be excluded from analysis, an approach used elsewhere (Conroy, 2017; Newnham, McKellar, & Pincombe, 2017). No individuals opted out.

### Within-Case Methods: Data Collection

#### Interviews

Semistructured topic guides were designed to provoke discussion of perceptions of the residents’ identity over their life course, their transition to the care home, and subsequent adjustment. Staff were asked for their perspectives on their roles in promoting identities within the care home, on perceived barriers and facilitators, and on residents’ adjustment over time. Questions included “Tell me about your move to the care home,” “What would you consider to be a ‘good’ day for you?” (Residents), “How would you describe [the resident]?”, “What would you consider to be meaningful activities for him/her?” (Relatives), “Tell me about a time a resident moved here,” “How do you incorporate individuality within the care home?” (Staff). Questions were developed iteratively to reflect emerging topics and themes.

#### Observations

All residents, staff, and relatives were eligible for inclusion in observations. Observations were exploratory and guided by SIP’s assertion that identities are influenced by social interactions and can be expressed externally, such as via hobbies and possessions. Observations and field notes focused on daily events in the care homes, including organized activities, daily care, and interactions between residents, staff, and visitors. Field notes also included conversations between participants and the first author. Residents without capacity to consent or opt-out were included in field notes for contextual purposes if they were central to observations involving other participants. Observations were a mixture of participatory and nonparticipatory: At times, the researcher remained a passive observer, but where possible she contributed informally to the daily life of the care homes by helping to serve meals and make drinks. This facilitated immersion in each care home and being allowed to witness personal care, such as dressing, an approach used in a similar context (Næss et al., 2016). Observations occurred on different days and times of day, including evenings and weekends, to reduce the possibility that data were focused around particular activities or participants.

Data collection ceased once data saturation was reached, where no new findings emerge in subsequent data collection, within or across cases (O’Reilly & Parker, 2012).

### Data Analysis, Rigor, and Validity

Transcripts of recorded interviews and field notes were managed using NVivo and analyzed, within and cross-case, using framework analysis (Ritchie & Spencer, 1994). This is a systematic and rigorous approach consisting of five interrelated stages (see Box 1), whereby iterative data collection and analysis of multiple data sources produce a transparent audit trail, so findings and interpretations are grounded in the data (Gale, Heath, Cameron, Rashid, & Redwood, 2013; Ward, Furber, Tierney, & Swallow, 2013). The theoretical propositions derived from SIP (section Study Design) informed the preliminary coding framework, which was continuously reviewed in light of emergent data-driven codes and themes. Pattern-matching of data against a priori propositions reconciles the diverse perspectives of a phenomenon within and across cases (Almutairi, Gardner, & McCarthy, 2014). Analysis generated a final analytic framework of 62 codes, grouped, and charted into four themes.

**Box 1. Stages of Framework Analysis**

1. **Familiarization**
  Immersion in the data. Read complete transcripts and field notes.
2. **Identify a thematic framework**
  Initial development of a coding framework developed through a priori issues and familiarization stage.
3. **Indexing**
  The process of systematically applying the thematic framework to data. Changes made as necessary to reflect the data.
4. **Charting**
  Using headings from thematic framework to create charts of data.
5. **Mapping and interpretation**
  Searching for patterns and explanations in the data.

All data were collected by the first author (K. Paddock), who has prior experience working and researching in social care settings, but is not a clinician. She led data analysis, and regularly discussed emerging findings and experiences with the other three authors, two of whom (C. Brown Wilson and C. Walshe) are registered nurses with experience of working and researching in residential and social care.

Rigor and validity were ensured through the triangulation of multiple modes of data collection and sources of evidence across multiple cases, conducted over time. For respondent validation, the first author provided oral summaries of data and interpretations to participants, and invited comments.

To ensure reflexivity, the first author kept a reflexive diary alongside field notes to record her possible biases and role in shaping encounters. Developing a reflexive, iterative process between data collection and analysis continuously connected the data with emerging insights, leading to a more refined, and credible, understanding of identity (Lincoln & Guba, 1985; Shenton, 2004; Srivastava & Hopwood, 2009).

### Ethics

Research Ethics Committee approval was obtained from the University of Manchester and Northampton NRES committee (reference number 12/EM/0431). All names have been changed to pseudonyms to protect anonymity. Permission was only granted by the ethics committees to interview residents with capacity to consent.

## Results

Semistructured interviews were conducted with 18 participants, and over 260 hr of observations were conducted over a 12-month period across the three cases (see Table 2). Interviews lasted between 18 min and 1.5 hr. The majority of residents across the care homes had severe dementia, so could not be interviewed. Some participants were intimidated by a recorded interview, and many staff were too busy, so preferred discussions to be included as field notes (see Table 2). Residents also received very few visitors during the data collection period.

**Table 2.** Interview Sample and Observational Data Across Care Homes

	Care Home 01	Care Home 02	Care Home 03
Interviews	3 residents	4 residents	2 residents
	2 relatives	1 relative	1 relative
	2 staff	3 staff	0 staff
Observations	137 hr	84 hr	40 hr
Conversations during observations (not audio recoded)	8 residents	8 residents	4 residents
	1 relative	3 relatives	0 relatives
	7 staff	5 staff	4 staff

Within-case analysis generated substantially similar experiences and themes in each care home, and thus, results from a cross-case analysis are presented, with any divergent themes discussed. The four interrelated themes are as follows:

1. Changing with age, and how this predated a move into a care home;
2. Bounded identity;
3. Independence and autonomy; and
4. Social comparisons.

### Changing With Age

Prior to the relocation to a care home, residents and relatives acknowledged that increased frailty impeded residents’ abilities to perform everyday tasks and meaningful activities, which influenced their self-perception.

Ageing is a terrible thing . . . You can’t do what you used to do . . . (Hayley [resident], interview, Care Home 03)

Social networks and interactions gradually receded due to bereavements or family and friends moving away, which made residents feel disconnected and unable to be themselves:

I miss my people. Where are my people? They know who I am . . . (Philippa [resident], field notes, Care Home 02)

Residents adapted their homes, hobbies, and activities to accommodate these changes. For instance, Ruth (resident, Care Home 02) connected with her family and friends by knitting items for them, but her arthritis restricted her ability to hold knitting needles, so she began crocheting, which uses a different type of needles. This enabled Ruth to continue to make gifts and maintain a feeling of connectedness to important social networks.

The aging process had affected residents’ sense of self, but some had been able to employ strategies to help mitigate its impact. The care home further impeded their established identities and restricted residents’ abilities to adjust in a manner most acceptable to themselves.

### Bounded Identity

Residents’ own homes served as a benchmark for the expression of their personal identities, particularly through possessions, clothing, and activities, but the care home environment largely restricted this.

### Possessions

All participants agreed that personal possessions helped residents to express their personal and social identities and served as anchors to important memories.

When they wake up ‘til they go to sleep they have that sense of belonging. That this is my room now . . . I know that I bought that clock at such and such a place . . . and that picture there of my husband, that’s a reminder of me and my husband when I was younger . . . (Charlotte [staff, manager], interview, Care Home 02)

Staff emphasized that rooms could be personalized with furniture from home, but there was limited scope to do so because of the small size of most bedrooms. Residents had to relinquish many personal possessions, which upset them and their families, as this was associated with loss of important memories and symbols of identity. Julia (Care Home 01) had been a seamstress, her sewing machine a symbol of her independence, and an anchor for memories of her deceased husband. It was too large for the care home, and its loss signified the loss of important identities and memories:

. . . I’ll never operate the sewing machine again. It’s just the fact that [it’s in storage, not with her]. And it’s my past. (Julia [resident], interview, Care Home 01)

Residents across the care homes had little opportunity to acquire new possessions because there were infrequent visitors to support procurement. Staff typically focused on the occasional acquisition of practical items, such as underwear or toiletries. However, staff also stated that the minimal involvement of relatives made it difficult for them to learn about the preferences of residents with less communicative ability. There were anomalous instances where staff purchased meaningful items for residents, such as a stereo for a resident who loved music (Care Home 01), and jewelry in the color of a resident’s favorite football team (Care Home 02).

### Clothing

Residents and relatives often mentioned the importance of personal aesthetic. Residents admitted to the care home as an emergency had little input into which belongings they kept or relied on clothing borrowed from other residents or purchased by staff. Clothing was occasionally lost or mixed-up between residents, which upset residents and their relatives, who felt that an element of themselves had been stolen. This was particularly pertinent for relatives of residents with dementia, as they felt it highlighted their increased depersonalization and powerlessness:

It was like she was wearing part of me mum. (Amanda [relative], field notes, Care Home 02)

As care needs increased, staff in Care Homes 01 and 02 in particular tended to dress residents in looser-fitting, easy-to-change and easy-to-clean clothes, or “babywear” (Twigg and Buse, 2013: 330), regardless of the individual’s personal aesthetic. However, there were notable examples across each care home of staff making an effort to incorporate residents’ preferences in their daily care, typically in relation to colors, or whether someone was a “skirt person” or a “trouser person”:

. . . Joanna [staff], said that it’s ‘a bit of a faff’ getting them in and out of trousers, “but it’s what they prefer” . . . (Field notes, Care Home 03)

### Activities

Residents derived a sense of self through their hobbies and activities. Staff in each care home initially claimed to incorporate residents’ preferences, but during observations, this rarely occurred. Staff felt constrained by understaffing and limited resources and unable to support residents’ identities and individuality. Consequently, there were few activities overall, and observed activities were based on generalizations to please the most people and did not account for nuanced preferences. These included a music-themed reminiscence group, tai chi (Care Home 01), bingo, and a “memory man” who discussed local history (Care Home 02):

. . . let’s say someone’s gay, and like to go to gay bars, and would like to meet gay people, erm, for example. Um, or let’s say someone’s Caribbean and they like to go to Caribbean clubs . . . I find they kind of take the headline title [of residents’ preferences] and that’s about it. (Adam [staff], interview, Care Home 01)

This approach did not satisfy most residents and relatives, who complained about a lack of stimulation and false promises of individualized activities.

I mean, Tracey [manager] said that they did lots of things in the afternoon, and I’ve never been convinced they’ve done as many as Tracey said they did. (Daniel [relative], interview, Care Home 01)

Residents and relatives acknowledged the financial constraints of many care homes, but felt more could be done to improve daily life. Staff also highlighted difficulties of organizing activities for residents with physical and cognitive impairments:

. . . It’s hard to think of where they can go really. You got to think about where they’re going to go to the toilet and everything—so there’s loads to think about before you even take them out. (Laura [staff], interview, Care Home 02)

Participants in Care Home 03 mentioned plans of a daytrip, but none occurred during the data collection period. However, in Care Home 03, some residents attended a weekly church fete unchaperoned; a luxury they valued. Across all three care homes, television was the most common activity observed. There were limited opportunities for residents to suggest ad hoc activities beyond the immediate resources of the care homes.

### Independence and Autonomy

Residents and their relatives frequently emphasized the importance of independence as an element of residents’ identities throughout their life course, and evidenced this in a variety of ways. For instance, Carrie (Care Home 02) was an international fashion buyer; Mary (Care Home 02) attended football matches “with the boys,” which was considered unusual at the time; Richard (Care Home 03) was a freelance photographer.

Repeated reflections on their independence highlighted its absence in the care home. Residents missed the freedom to set their own agendas for the day. The care homes all adopted similar routines: set times for waking residents and putting them to bed, for food and drink, and any activities. Staff discouraged deviations from these routines as it jeopardized the smooth running of the care home. Residents felt that minor changes to routines were occasionally catered for, but at a compromise; Louis (resident, Care Home 03) had requested to sleep in one day, but was then allegedly denied his breakfast as the allotted breakfast time had passed and staff were busy elsewhere. Participants’ perceptions of how successful these allowances and compromises were in practice differed:

Well Ruth (resident) likes to get up really early—Ruth likes to get up at like quarter to seven. . . . So—like when they first come [to the care home], you ask them, like what they like to do . . . (Laura [staff], interview, Care Home 02)

I like to get up early. But I have to wait for the nurse [to get me up]. (Ruth [resident], interview, Care Home 02)

Strict health and safety policies and organizational efficiency meant risk-averse staff tended to complete minor tasks themselves, such as making hot drinks, which undermined residents’ independence. The role of staff as carers seemed at odds with the expectation that they should also facilitate independence, particularly because of limited resources:

. . . It’s all well and good saying they want to remain independent, but if you can’t walk, you can’t walk . . . It is our job at the end of the day—to keep them well . . . (Edna [staff], field notes, Care Home 02)

To counter the negative perceptions of aging and increased dependency, many residents amended their definitions of independence and autonomy to emphasize minor daily tasks and accomplishments. Autonomy within the care home was limited to small day-to-day decisions, such as choosing a meal from the available selection, requesting an alternative meal where possible, or deciding when to go to bed if they were physically able to do so unaided. Physical independence to perform certain small tasks such as setting tables at mealtimes, usually authorized by staff, helped residents to feel as though they had retained an important element of their personal identities:

Catherine . . . helped to place the cutlery on the tables . . . and added “I know I’m not completely independent anymore. But it’s something” . . . (Catherine [resident], field notes, Care Home 01)

Each care home had members of staff who made a conscious effort to accommodate residents’ autonomous decision making and individual preferences. A notable example involved Edna’s (staff, Care Home 02) determination to allow a resident a “duvet day,” who uncharacteristically wanted to stay in bed. These infrequent instances encouraged residents to express their individuality, and made them feel more in control of their surroundings and their care. With residents’ new perceptions of independence and autonomy largely based on physical capabilities, this enabled them to use levels of cognition as a source of comparison against other residents who experienced dementia or mental health problems.

### Social Comparison

Residents without dementia accepted that moving to a care home was necessary because of their care needs, but felt their positive sense of identity was jeopardized because of the association of care homes with severe cognitive and physical impairment. Residents with dementia represented these negative stereotypes, and symbolized the worst aspects of aging. Consequently, residents without dementia distanced themselves from residents with dementia by engaging in social comparison. They regularly pointed out those with dementia and emphasized their own perceived cognitive superiority, while also expressing sympathy and frustration over the often repetitive or disruptive behaviors associated with severe dementia. Such downward social comparisons serve to enhance self-image and in turn improve well-being (Gibbons & Gerrard, 1991):

[Philippa] was looking at the row of residents sat asleep against the wall . . . “Most of these have lost their minds, you know . . . I can still think for myself. I haven’t gone yet” . . . (Philippa [resident], field notes, Care Home 02)

Residents without dementia typically vocalized their comparisons with members of staff, visitors, or the researcher; not with one another. Only two residents in Care Homes 01 and 03 indicated that they were friends and regularly conversed. Most residents suggested they were lonely, but did not converse with others beyond mealtimes. Although residents stated they were unable to hold meaningful social interactions with residents with dementia, it was difficult to determine why residents without dementia did not engage more with one another. Some participants, particularly staff, suggested residents may not wish to invest in making connections with others because they are acutely aware of their own mortality. When asked, residents said that they simply did not like the other residents, or that it was a lot of effort, especially if they were at risk of developing dementia.

I asked Elizabeth why she didn’t chat to Carrie more . . . after they seemed to have a nice time the other day . . . Elizabeth pulled a face and after a pause said “she’ll probably end up like the rest of ‘em in here . . .” (Field notes, Care Home 02)

## Discussion

The purpose of this study was to explore how life in a care home affects on the identities of care homes residents. The use of SIP offers a broad approach to identity that highlights the importance of context-bound social interactions for the development and maintenance of identity within the unique context of a care home. At the outset of the study, we proposed three theoretical propositions. In relation to these propositions, our findings reveal that (a) residents renegotiate their identities within the context of the care home, but use social interactions to facilitate social comparison with more impaired individuals, while largely failing to establish new relationships; (b) care homes have the potential to accommodate a multitude of identities by facilitating links with previous social networks or symbols that are necessary to maintain a sense of self, (c) but lack adequate support or appropriate resources to achieve this. We now discuss these findings in detail, followed by their implications.

Findings confirm the role of activities, possessions, and clothing, in symbolizing identities, particularly in light of personal and physical loss. Continued identification with such meaningful symbols help to bolster identity, even for individuals with limited expressive capacity, and can be maintained through adapted ways of living (Black et al., 2018; Cohen-Mansfield, Marx, Thein, & Dakheel-Ali, 2010; Lloyd, Calnan, Cameron, Seymour, & Smith, 2014). But for participants in the present study, the care home environment undermined their abilities to adapt, disrupting connections to many important symbols, and resulting in a limited, bounded expression of residents’ identities.

Institutional restrictions, standardized routines, and strict risk management also threatened residents’ independence and autonomy, as perceived staff shortages and limited resources necessitated the precedence of organizational efficiency over individual needs. It has been established that a failure to satisfy needs for independence and autonomy is related to depressive symptoms and poor well-being and also hinders individuality and the expression of personalities (Custers, Westerhof, Kuin, Gerritsen, & Riksen-Walraven, 2012; Goffman, 1961; A. Haslam et al., 2009; Knight et al., 2010; Wiersma & Dupuis, 2010; Kloos, Trompetter, Bohlmeijer, & Westerhof, 2018). Consistent with other studies (Falk, Wijk, Persson, & Falk, 2013; Golander, 1995; Wiersma & Dupuis, 2010), participants in the present study reported an “emotional limbo” between the awareness of residents’ increased care needs and dependency on staff, and the importance of independence to residents’ identities. Our findings show that this motivates residents to emphasize their physical abilities to perform small tasks, to reconcile their established identities within a new, more constrained context.

Maintaining links with social networks or establishing new identity-relevant connections is also necessary to reinforce a sense of self and to buffer against a threatened identity or well-being (Cohen-Mansfield, Golander, & Arnheim, 2000; A. Haslam et al., 2009; Surr, 2006). Relatives have the potential to support residents’ identity and improve resident outcomes by maintaining relationships with them and with staff (Davies & Nolan, 2006; Roberts & Ishler, 2017), but most residents in the present study had little contact anyone outside of the care home and thus were unable to maintain identity-affirming connections. However, residents did not appear to value the opportunity to develop friendships with one another, as has been described elsewhere (Surr, 2006; Tester et al., 2004). Rather, our findings resonate with those of Abbott and colleagues (2017), where residents cited various barriers to social interactions with others, but participants in the present study focused on cognitive impairment as the fundamental obstacle. The fact that most older people residing in the care homes of the present study had a diagnosis of dementia reflects the national statistics of the United Kingdom on levels of impairment in care homes (Alzheimer’s Society, 2014). But the belief among unimpaired residents that residents with dementia, particularly those who also had severe physical impairments, were not viable companions and should be avoided, reflects a more complex issue relating to threatened identities.

According to SIP, psychological strategies such as social competition, social creativity, and social mobility can be used to protect a threatened identity (Reicher et al., 2010; Tajfel, 1981; Tajfel & Turner, 1979). The present study has shown that by highlighting the impairments of others and emphasizing their own abilities, residents without dementia used social creativity, specifically social comparison, as a means of cognitive adaptation. With little consistent opportunity to buffer identities through other means, social comparison and the motivation to distance oneself from impairment may have also served to alienate residents from one another who could have formed meaningful relationships, whether cognitively impaired or otherwise.

### Implications

Although global policy states that care provision should enable self-expression and identity, the ability to make choices, and to maintain connections with social networks (World Health Organisation, 2015), our findings suggest that such goals are difficult to achieve in the care home setting. Few visitors and opportunities to maintain connections outside of the care home place greater pressure on staff to perform identity work. To move forward, we need to understand how guidelines or training on identity is implemented in care homes and how this may be improved. In addition, further research on how residents can maintain connections outside of the care home is needed.

Staffing and resource constraints cannot be easily rectified, but care homes could facilitate residents’ needs within the allowances of their means. This study has demonstrated the value of seemingly minor, but meaningful, interactions between stakeholders, small changes to routines, and supported independence and autonomy. Evidence suggests that interventions to facilitate group-based decision making among care home residents regarding the refurbishment of communal areas created a shared identification, increased social engagement, and improved cognitive function and life satisfaction (C. Haslam et al., 2014). Future interventions could support residents to make collective decisions in other, smaller areas of care home life, such as weekly activities or menu choices. This can promote positive social interaction among stakeholders and improve feelings of independence and autonomy, thereby minimizing the necessity for some residents to distance themselves from others. An assessment of such interventions versus standard practice, focusing on the cost implications and impact on staff workload, can help to determine their feasibility in under-resourced facilities.

### Strengths and Limitations

This study is the only U.K.-based study to date that has used observational and interview methods across care homes with SIP. A key strength is the volume of data collected: Over 260 hr of observations across 1 year, combined with iterative interviews, facilitated in-depth exploration of context-bound data to understand the phenomenon of identity management over time. SIP has contributed to other social care areas, and its use in the care home setting helped to uncover and explore psychological strategies used by residents to cope and maintain a positive sense of self.

We only interviewed residents who had capacity to consent, which limits the generalizability of the findings. Future observational studies are needed that includes the perspectives of all care home residents. Furthermore, recruitment of care homes proved difficult. Managers were concerned with the potential distraction for staff or suggested the study had duplicitous aims in light of contemporaneous negative media representations of care homes. Although sampling was purposeful, no purpose-built care homes agreed to participate. There were also very few visitors across each care home, resulting in a small sample of relatives interviewed. Although this may limit generalizability, it is nonetheless an artifact of each case and serves to highlight the contemporaneous issues of maintaining relationships, and in turn identity, in care homes.

## Conclusion

This study explored how life in a care home affects residents’ identities. The use of SIP within a multiple case study design, with interview and observational methods, is unique in care home research. Although moving to a care home initially emphasized age-related changes, institutional restrictions and limited social networks further undermined residents’ identities over time. The use of social comparison by residents without dementia served to buffer against daily threats to identity, in particular, the threat of being considered severely cognitively impaired and lacking independence. Resource constraints can make it difficult to adequately support diverse identities, yet even small changes to routines and daily care can help. Adequate resources and support within care homes can facilitate the expression of positive identities. This may reduce the need for staunch social comparison and create a more constructive environment for all residents, which may in turn improve well-being.

## Funding

This study was funded by Medical Research Council doctoral studentship award for K. Paddock. Grant reference MR/J500410/1.

## Conflict of Interest

None reported.
